# Extensive lesions and a positive cone margin are strong predictors of residual disease in subsequent hysterectomy following conization for squamous intraepithelial lesion grade 2 or 3 study design

**DOI:** 10.1186/s12905-023-02568-w

**Published:** 2023-08-28

**Authors:** Hongfa Peng, Wencan Liu, Jingjing Jiang, Hui Du

**Affiliations:** 1 Department of Obstetrics and Gynecology, Second hospital of Hebei medical university, Shijiazhuang City, 050000 Hebei China; 2 Department of Obstetrics and Gynecology, Hebei General Hospital, Shijiazhuang City, 050051 Hebei China

**Keywords:** Cervical intraepithelial neoplasia, Cervical conization, Residual lesions, Margin involvement, Extensive lesions

## Abstract

**Background:**

This study aimed to determine the predictive factors for post-conization of residual disease in subsequent hysterectomy for cervical intraepithelial neoplasia grade 2 or 3.

**Methods:**

This retrospective study included 267 patients with histologically confirmed cervical intraepithelial neoplasia grade 2 or 3 who underwent hysterectomy within 7 months after conization. Clinical data (e.g., age, menopausal status, gravidity, parity, type of transformation zone, conization method) as well as pathological data pertaining to conization and hysterectomy were collected from medical records. A logistic regression model was used to analyze the relationship between the variables and risk of residual lesions in hysterectomy samples.

**Results:**

Overall, 70 (26.2%) patients had residual lesions in their hysterectomy specimens. Univariate analyses revealed that age ≥ 50 years (*p*=0.019), endocervical gland involvement(*p*=0.013), positive margin(*p* < 0.001), and involvement of 3–4 quadrants(*p* < 0.001) were risk factors for residual lesions. Conversely, postmenopausal status, gravidity ≥ 3, parity ≥ 2, loop electrosurgical excision procedure, and type III transformation zone were not risk factors for residual lesions. A positive margin(*p* < 0.001) and multiple-quadrant involvement(*p* < 0.001) were identified as independent risk factors for residual lesions on multivariate analysis.

**Conclusions:**

Multiple-quadrant involvement and a positive cone margin were reliable predictive factors for residual disease. Total hysterectomy or repeated cervical conization should be considered for patients with these two risk factors. The identification of high-risk patients with extensive lesions by colposcopic examination before conization is indispensable, as it will enable surgeons to perform conization with consideration of risk factors and possibly improve the approach used for the excisional procedure. For high-risk patients, colposcope-guided cold-knife conization is preferred when resources permit.

**Supplementary Information:**

The online version contains supplementary material available at 10.1186/s12905-023-02568-w.

## Background

Therapeutic cervical conization is an effective method for treating women with cervical intraepithelial neoplasia grade 2 or 3 (CIN 2/3) [1, 2]. Nonetheless, 20–25% of women develop residual lesions after surgery, which represents a common limitation of conization [3]. Women with incompletely excised CIN 2/3 are at increased risk for residual/recurrent CIN 2/3 or worse [4, 5]. The identification of patients with residual lesions can facilitate the treatment of residual CIN 2/3 or invasive carcinoma without delay and avoid overtreatment resulting from the secondary excision of uninvolved tissues. Therefore, accurate prediction of residual disease following conization is of utmost importance for the follow-up management of patients with CIN 2/3, and predictors of residual lesions that may enable the identification of correct treatment for each patient must be determined. Several researchers have investigated risk factors potentially associated with residual CIN 2/3 and have recognized margin involvement, lesion size, severity and location, depth of conization, conization method, older age, menopausal status, and the presence of high-risk human papilloma virus after conization as factors that may aid in identifying women at the highest risk for residual lesions [5–11]. Currently, there exists considerable debate as to which of these factors, or a combination of factors, most accurately predicts residual lesions. A more accurate predictor of residual lesions after conization is urgently needed, particularly in women of child-bearing age. Hence, the present study aimed to determine the predictive factors for post-conization residual lesions in subsequent hysterectomy for CIN 2/3.

## Methods

The retrospective study protocol was approved by the Institutional Review Board of the Second Hospital of Hebei Medical University. The requirement for patient consent was waived by the Board because of the retrospective nature of the study. This study conforms to the provisions of the Declaration of Helsinki [12]. This study included patients with histologically confirmed CIN 2/3 who underwent cold-knife conization (CKC) or loop electrosurgical excision procedure (LEEP) and then hysterectomy within 7 months, irrespective of margin status, between January 2018 and January 2022 at the Department of Gynecology in the Second Hospital of Hebei Medical University. For performing excision without colposcopy assistance, initially, the cervix was swabbed with Lugol’s iodine solution to assist in locating the ectocervical margin of the lesion. After delineating the area of abnormality with Lugol’s iodine solution, a circular knife cut approximately 0.5 cm outside the area not stained with iodine was made using a pointed- and angled-cold knife. Clinical data (e.g., age, menopausal status, gravidity, parity, type of transformation zone [TZ], conization method) as well as pathological data pertaining to conization and hysterectomy, including the grade of CIN, margin status, and endocervical gland involvement (EGI), were collected from medical records. The margins of the specimens included the endo- and ectocervical margins. Residual lesions were defined as the presence of CIN 2/3, invasive cancer, or adenocarcinoma in situ in hysterectomy specimens. Women with incomplete clinical data were excluded from the analysis.

### Statistical analysis

Discrete and categorical variables are expressed as median (range) and numerical (percentage) values, respectively. A univariate logistic regression analysis was conducted to assess predictive factors for residual lesions in patients with CIN 2/3 after hysterectomy following conization. For comparison, we used cutoff values of ≥ 50 years for age, 3 for gravidity, and 2 for parity. Additionally, we semi-quantitatively described the lesion size as quadrant(s) of the cervix involving CIN lesion. Patents were divided into groups according to the number of quadrants showing disease involvement: patients with one or two and these with three of four quadrants. All risk factors associated with residual lesions in the univariate logistic regression analysis were analyzed using multivariate logistic regression. Considering that we included women with no follow-up losses, sample size calculation was performed using the estimation of a confidence interval (CI) with a required width for a single proportion based on the primary outcome: odds ratio (OR) of residual CIN 2/3 or worse in patients with positive and negative margins. The literatures report a range of OR between 2.43 and 4.8 [3, 5, 13, 14], and we expected a mean value of 3. With a confidence level of 95% and CI width (2-sided) equal to 10 (± 5%), the minimum required sample size was determined to be 152 women. Statistical analysis was performed using SPSS for Windows version 19.0 (SPSS Inc., Chicago, IL, USA). Univariate and multivariate logistic regressions were used to calculate the ORs and 95% CIs after simultaneously controlling for potential confounders. All *p-*values were two-tailed, with statistical significance set at *p* < 0.05.

## Results

### Clinical characteristics of the patients

A total of 267 patients (median age: 48.0 years [range: 29–77 years]) were included in the analysis of residual lesions. The median interval from conization to hysterectomy was 5 days (range: 1–192 days). Within the time interval between these two operations, six patients underwent a high-risk human papillomavirus test and pap smear. Two patients underwent colposcopic examination and cervical biopsy. Among the patients, 134(50.2%) were postmenopausal. The median gravidity and parity were 3.0 (range: 0–10) and 2.0 (range: 0–5), respectively. Type I or II TZ was observed in 210 patients (78.7%). LEEP and CKC were performed for therapeutic conization in 8.6% and 91.4% of the patients, respectively. Clinical characteristics of the patients are summarized in Table 1.

**Table 1:** Baseline characteristics of all 267 patients

Characteristics		Parameters	Values (%)
Age		Median	48
		25th -75th percentile	42–53
		20–29	1(0.4%)
		30–39	44(16.5%)
		40–49	126(47.2%)
		50–59	79(29.6%)
		60–69	16(6.0%)
		>70	1(0.4%)
Gravidity		Median	3
		25th-75th percentile	2 ~ 4
Parity		Median	2
		25th-75th percentile	1 ~ 2
Menopausal status		Premenopausal	133(49.8%)
		Postmenopausal	134(50.2%)
Transformation zone		type I	125(46.8%)
		type II	85(31.8%)
		type III	57(21.3%)
Type of treatment procedure		CKC	244(91.4%)
		LEEP	23(8.6%)
Surgical margin status		Positive	83(31.1%)
		Negative	184(68.9%)
Residual disease		Positive	71(26.6%)
		Negative	196(74.4%)

LEEP, loop electrosurgical excision procedure; CKC, cold-knife conization; TZ, transformation zone

### Pathological characteristics of conization specimens and corresponding residual lesions rates

The pathological results of conization indicated that EGI was present in 175 (65.2%) patients, and 83 (30.7%) of them had a positive resection margin. Of the 83 cases, 30 had positive endocervical margin, 44 had positive ectocervical margin, and 9 had both positive endocervical and ectocervical margins. Following hysterectomy, 197 (73.8%) patients exhibited no evidence of CIN 2/3, whereas 3 (1.1%) patients had previously undiagnosed cervical cancer (2 patients with adenocarcinoma in situ [AIS] and 1 with microinvasive carcinoma). The two patients with AIS were postmenopausal women. Their pre-cone human papillomavirus (HPV) testing results suggested high-risk HPV positivity, and the pre-cone cytology results of one woman suggested atypical squamous and atypical glandular cells. Pathological examination of the uterine specimens of both patients showed HPV-related adenocarcinoma. Residual lesions, including CIN 3 (*n* = 40), both CIN 3 and EGI (*n* = 15), and CIN 2 (*n* = 12), were detected in hysterectomy specimens of 67 (25.1%) patients. Additional details are provided in Table 2.

**Table 2:** Association between pathological characteristics of conization specimens and residual disease

Characteristic	Parameter	Number of patients (n = 267)		
		Total n		Residual lesion n (%)
Margin status n (%)	Positive	83		45(54.2%)
	Endocervical margin positive	30		19(63.3%)
	Ectocervical margin positive	44		24(54.5%)
	Combine margin positive	9		9(100%)
	Negative	184		25(13.6%)
Glandular involvement n (%)	Involved	194		55(28.4%)
	Not involved	73		11(15.0%)
Quadrants involvement n (%)	1 quadrant involved	50		1(2.0%)
	2 quadrants involved	49		8(16.3%)
	3 quadrants involved	50		11(22.0%)
	4 quadrants involved	118	50(42.4%)	

### Univariate and multivariate analyses for the prediction of residual lesions

Univariate logistic regression analysis revealed that a positive margin, multiple-quadrant involvement, EGI, and age ≥ 50 years were all associated with residual lesions (Table 3). In contrast, other parameters such as menopause, gravidity, parity, type of TZ, and conization method had no predictive value for residual lesions. Subsequently, we used multivariate logistic regression to analyze positive margin, multiple-quadrant involvement, glandular involvement, and age ≥ 50 years. Multivariate analysis showed that a positive margin and multiple-quadrant involvement were risk factors for residual lesions (*p* < 0.05; Table 4). A logistic regression analysis revealed that involvement of the four quadrants was the only significant independent predictor of residual lesion (Additional File 1). The risk of residual CIN 2/3 or worse varied according to the anatomical localization of the margin (endocervical margin: OR, 10.985[95% CI, 4.677–25.804]; ectocervical margin: OR, 7.632[95% CI, 3.685–15.805]; for both endo- and ectocervical margins, residual lesions were found in all nine patients). We also conducted a univariate logistic regression analysis to assess predictive factors for a positive margin in patients with CIN 2/3 prior to therapeutic cervical conization. We found that postmenopausal status (OR, 2.7), age ≥ 50 years (OR, 0.44) and parity 2 or more (OR. 2.6) were associated with positive margin (Additional file 1).

**Table 3:** Univariate analyses for demographic and clinicopathological parameters related to residual disease in post-conization hysterectomy specimens

Parameter	Numbers (n = 267)		OR (95%CI)	*P*-values
	Total n	RD n (%)		
Age				
≥50	96	17(17.7%)	2.087	
<50	171	53(31.0%)	1.127–3.867	0.019
Menopause				
YES	134	41(30.6%)	1.581	
NO	133	29(21.9%)	0.911–2.745	0.104
Gravidity				
≥3	193	51(26.4%)	1.04	
<3	55	19(34.5%)	0.564–1.917	0.901
Parity				
≥2	199	54(27.1%)	1.21	
<2	68	16(23.5%)	0.637–2.299	0.56
Conization method				
LEEP	23	8(34.8%)	1.566	
CKC	244	62(25.4%)	0.633–3.871	0.332
Glandular involved				
Positive	194	59(30.4%)	2.463	
Negative	73	11(15.1%)	1.211–5.013	0.013
Type of TZ				
Type III	57	12(21.1%)	0.655	
Type I-II	210	58(27.6%)	0.325–1.321	0.237
Quadrants involved				
≥3	168	61(36.3%)	6.271	
<3	99	9(9.1%)	2.958–13.294	<0.001
Margin status				
Positive	83	45(54.2%)	7.532	
Negative	184	25(13.6%)	4.119–13.772	<0.001
Ectocervical margin status				
Positive	44	24(54.4%)	7.632	
Negative	184	25(13.6%)	3.685–15.805	<0.001
Endocervical margin status				
Positive	30	19(63.3%)	10.989	
Negative	184	25(13.6%)	4.677–25.804	<0.001

RD, Residual disease; OR, odds ratio; LEEP, loop electrosurgical excision procedure; CKC, cold-knife conization; TZ, transformation zone

**Table 4:** Multivariate analyses for demographic and clinicopathological parameters related to residual disease in post-conization hysterectomy specimens

Variables	B	S.E	Wals	Sig	Exp(B)	95%CI
Margin positive	1.625	0.33	24.309	<0.001	5.079	2.662–9.69
Glandular involved	0.051	0.444	0.13	0.909	1.052	0.441–2.512
Age ≥ 50	-0.272	0.352	0.597	0.44	0.762	0.382–1.519
Multiple quadrants involved	1.055	0.457	5.339	0.021	2.783	1.174–7.032

## Discussion

The present study aimed to determine the predictive factors for post-conization residual lesion in subsequent hysterectomy for CIN 2/3, and we confirmed that margin involvement and extensive lesions were predictors of residual lesions in hysterectomy specimens. As reported by previous studies, the probability of residual lesions ranges 30–90% if the resection margin of a cone specimen is positive [3]. Moreover, women with margin involvement have a five times higher relative risk of residual or recurrent CIN 2/3 than that of women with negative margins [3]. Thus, margin status is generally considered as a predictive factor for residual lesions [5, 11, 15–18]. Specifically, the endocervical margin status has been recognized to be significant, whereas the exocervical margin status remains controversial [18–23]. The majority of our patients were of reproductive age and perimenopausal, and 210(78.7%) patients had either type I or II TZ. The most involved margin in our patients was the ectocervical margin. Our analysis indicated that the risk of residue increased with cervical margin involvement, irrespective of whether the endocervical or ectocervical margin was involved. Moreover, our study showed that involvement of both endo- and ectocervical margins was more likely to result in residual lesions than the involvement of endocervical or ectocervical margins alone. However, the specificity of our results might be related to subject bias, choice of surgical approach, excision without colposcopy assistance, and short interval between conization and hysterectomy.

In this study, the rate of positive margin after conization was higher compared with that reported in some previous studies [3, 5], especially the ectocervical margin. This may be related to the following factors. First, we performed excision without colposcopy assistance in our study. Currently, colposcopy-guided excision is the standard of care for women with CIN 2/3 in developed countries. However, where resources do not permit, some patients with CIN 2/3 are still being treated with conization without colposcopy assistance. In such circumstances, identification of high-risk patients with extensive lesions by colposcopic examination before conization is indispensable. Second, in our institution, CKC is performed as the definitive treatment of CIN 2/3. Patients treated with CKC have been shown to have lower rates of positive endocervical margin than those treated with large loop excision of the TZ [24]. Third, residual lesions at the edges of the cervix after conization may be eliminated by rapid cell turnover during cervical healing and by vaginal acidity [25]. However, in our study, the short time interval between the two operations limited the elimination of the lesions by rapid cell turnover. The results obtained by Cejtin et al. are consistent with our findings [22].

Margin involvement is regarded as an important predictor of residual disease [5, 14, 26]. Repeated cervical conization is considered an acceptable alternative for women with a positive cone margin who desire fertility preservation, and hysterectomy is the definitive therapy for women with no reproductive requirements [27, 28]. If the choice of treatment is new conization or hysterectomy, most women will unnecessarily undergo these procedures because they have no residual lesions. Such unnecessary surgery increases the risks for complications and affects these women’s gestational future. However, if surgery is not performed, there is a risk of insufficient treatment in a large number of women with CIN 2/3, as well as a risk of malignancy. This presents a problem for both patients and gynecologists when planning follow-up and further therapy. Therefore, it is necessary to distinguish the subset of patients with residual lesions from those with positive margins and identify risk factors associated with surgical margins in order to reduce or avoid positive margins.

Previous studies showed that HPV testing is an effective tool in predicting residual disease after conization [3, 14, 29]. The predictive value of resection margin for predicting residual disease improved when used in combination with the HPV test [3]. Positive excision margins and high-risk HPV infection at follow-up, appeared to be strong risk factors for residual/recurrent CIN 2/3 after conization [5, 13]. The combination was thus suggested for use in risk stratification for residual/recurrent disease [30]. Women with positive margins and high-risk HPV infection during follow-up should be considered for prompt re-treatment. However, the impact of HPV status on cervical glandular lesions is controversial [31–33]. Therefore, further research is needed to assess the role of the combined margin and post-excision HPV status in stratifying the risk of treatment failure and follow-up management. In this study, data on HPV status after conization was scarce because most patients underwent hysterectomy shortly after conization. Surprisingly, three patients with positive margins in our cohort were found to have a previously undiagnosed cervical cancer following hysterectomy. Two of the three had HPV-related adenocarcinoma. Women with incompletely excised CIN 2/3 are at risk for cervical cancer. One study found an incidence of micro-invasive carcinoma of 10.38% in the final histopathological analysis of hysterectomies performed for CIN 3 [34]. Patients with positive margins may be considered for repeated conization or hysterectomy [35].

Currently, hysterectomy is not advisable for treating CIN 2/3 [2]. However, the procedure is acceptable, after obtaining informed consent from the patients, only if it is not possible to carry out or repeat a diagnostic excision or if adequate follow-up is not feasible [2]. Despite this, 267 women with CIN 2/3 were treated with hysterectomy in our study, suggesting that the procedure is still commonly performed. Based on their records, in addition to the aforementioned factors, the following several factors led our patients to choose hysterectomy. First, some patients had other indications of hysterectomy, such as fibromatosis, adenomyosis, dysmenorrhea. Second, the CIN diagnosis and treatment caused anxiety and fear of cancer [36], leading them to seek a permanent solution through hysterectomy. Thus, it is important to address the patients’ anxiety and fears [37], which often stem from a lack knowledge regarding their conditions. Therefore, health care managers in primary and specialized care levels should create opportunities to meet patients’ informational needs. Third, limited healthcare access and financial concerns limited patients’ ability to perform self-care. Finally, the coronavirus disease pandemic radically changed China’s healthcare, also impacting screening and colposcopy programs. Hysterectomy is unacceptable as primary therapy solely for the treatment of CIN because it can lead to complications and risks of vaginal lesions onset and overtreatment [38]. Therefore, the choice of hysterectomy for CIN 2/3 should be carefully evaluated and considered only in selected cases.

Previous studies evaluated independent pre-conization variables and concluded that some pretreatment predictors might help in planning cervical conization [28, 39, 40]. In our study, logistic regression analysis revealed that involvement of the four quadrants was the only significant independent predictor of residual lesion. A univariate logistic regression analysis also showed that postmenopausal status, age ≥ 50 years and parity ≥ 2 were associated with positive margin in patients with CON 2/3 prior to therapeutic cervical conization. This finding is in agreement with previous results [40, 41].

Another significant finding of this study was that extensive involvement of CIN 2/3 at the cone margin (3–4 quadrants) was associated with residual lesions in subsequent hysterectomy specimens, which is consistent with previous results [24]. A previous study observed residual disease in 80% of patients with involvement of three or four cervical quadrants [42]. Women with extensive cone margin involvement (3–4 quadrants) were approximately 14 times more likely to have residual lesions on subsequent surgical evaluations [43]. Extensive lesions increase the incidence of residual lesions during conization because an increase in the range of lesions may affect observations, interfere with the judgment regarding surgical margins during the operation, and increase the surgical difficulty. Some researchers have suggested that the number of disease quadrants in a conization specimen can be used as an important factor in guiding subsequent treatment [44]. Repeat conization or hysterectomy is advised for women with the involvement of 3–4 quadrants. Nevertheless, post-conization surveillance without hysterectomy may be an alternative for women with involvement of 1–2 quadrants because they have a lower risk of residual disease. Correspondingly, the number of quadrants involved on colposcopic examination may serve as an assistive tool for assessing the size and shape of the excision [45]. For instance, Kawano et al. showed that cone lengths of 15 and 20 mm were the best cut-off values for the complete excision of cervical lesions involving a single quadrant and two or more cervical quadrants, respectively [46]. For the resection of large, scattered, and multifocal lesions, colposcopy-guided CKC may be more suitable because optimally excising large multifocal lesions using a round-loop electrode is difficult, as the latter results in circular excision, and the entire volume of a lesion may not be included in the excised circle.

In addition to positive margins and extensive cervical cone margin involvement, we suspected some variables to be associated with residual lesions; however, this suspicions were not proven. In particular, we found no significant differences in menopausal status, type of TZ, gravidity, parity, and conization method. Interestingly, age ≥ 50 years and EGI were associated with residual lesions on the univariate analysis, whereas a significant correlation for both was not observed on the multivariate analysis. The relatively small sample size and the heterogeneity of the patient population might have influenced our results. Age and menopausal status have been shown to be significant predictors of residual disease in several studies. This finding is anatomically plausible; atrophy of the genital tract and deep inversion of the TZ make the complete resection of the abnormal cervical epithelium challenging after menopause. However, neither of these factors was a risk factor for residual lesions in present study. This finding may be attributed to the patient heterogeneity. The majority of our patients were reproductive premenopausal women, among whom CIN was the most prevalent. In our study, 78.7% of patients had type I or II TZ, and the majority of patients had a positive ectocervical margin, which is anatomically plausible. Therefore, these factors should be evaluated further in future studies, and more accurate prediction of residual disease after conization is indispensable, especially in women of child-bearing age.

### Strengths and limitations

The strength of this present study was that the majority of our patients were in the reproductive and premenopausal periods, which are the main periods for the onset of CIN 2/3. Additionally, most patients underwent reoperation within a short period of time; hence, residual lesions were more accurately defined, and there was little likelihood of a new disease or regression. Moreover, we simultaneously included age, menopausal status, gravidity, parity, type of TZ, conization method, pathology of conization specimens (including the quadrants of lesions), and glandular involvement in a single study. Nonetheless, this study had some limitations, particularly its retrospective design, relatively small sample size, and heterogeneous patient population.

## Conclusions

In this study, we identified positive margins and extensive cone margin involvement as strong predictors of residual disease. Repeated cervical conization or total hysterectomy should be considered for patients with these two factors. In addition, extensive lesions increase the risk of a positive margin on cone specimens. The identification of high-risk patients with lesions involving 3–4 cervical quadrants before conization with consideration of risk factors and improve the approach used for the excisional procedure. For patients with extensive lesions, colposcope-guided CKC is preferred where resources permit.

### Electronic supplementary material

Below is the link to the electronic supplementary material.

**Additional File 1:** Table 1 and Table 2

## Data Availability

The datasets used and/or analyzed during the current study are available from the corresponding author on reasonable request.

## Abbreviations

CI: Confidence interval
CIN 2/3: Cervical intraepithelial neoplasia grade 2 or 3
CKC: Cold-knife conization
EGI: Endocervical gland involvement
LEEP: Loop electrosurgical excision procedure
OR: Odds ratio
TZ: Transformation zone
AIS: Adenocarcinoma in situ
