# Supplementary material for: Extensive lesions and a positive cone margin are strong predictors of residual disease in subsequent hysterectomy following conization for squamous intraepithelial lesion grade 2 or 3 study design
Source: BMC Womens Health. 2023 Aug 28;23:454. doi: 10.1186/s12905-023-02568-w (PMC10464471; doi:10.1186/s12905-023-02568-w)
Supplement: Supplementary file 1 — Additional File 1: Table 1 and Table 2 [file 12905_2023_2568_MOESM1_ESM.docx]

Supplemental Table 1 Demographic and clinicopathological parameters related to residual disease in post-conization hysterectomy specimens obtained from patients with margin involvement

| Variables | Category | Number (%) | *P*-value |
| --- | --- | --- | --- |
| Age | ≥50 | 6/17(35.3%) | 0.227 |
|  | ＜50 | 39/66(59.1%) |  |
| Menopause | Premenopausal | 14/28(50%) | 0.583 |
|  | Postmenopausal | 31/55(56.4%) |  |
| Gravidity | ≥3 | 35/59(59.3%) | 0.147 |
|  | ＜3 | 10/24(41.7%) |  |
| Parity | ≥2 | 36/68(52.9%) | 0.62 |
|  | ＜2 | 9/15(60.0%) |  |
| Conization method | LEEP | 6/8(75%) | 0.23 |
|  | CKC | 39/75(52.0%) |  |
| Glandular involvement | Positive | 40/72(55.6%) | 0.533 |
|  | Negative | 5/11(45.5%) |  |
| Type of TZ | Type III | 6/12(50%) | 0.751 |
|  | Type I-II | 39/71(54.0%) |  |
| Quadrants involved | ＞3 | 37/53(69.8%) | ＜0.001 |
|  | ≤3 | 8/30(26.7%) |  |
| Margin involvement | Ectocervical | 24/43(55.8%) | 0.149 |
|  | Endocervical | 12/31(38.7%) |  |

LEEP, loop electrosurgical excision procedure; CKC, cold-knife conization; TZ, transformation zone

Supplemental Table 2 Significant predictive factors for the positive margins

| Predictive factors | B | S.E | Wals | sig. | Exp(B) | 95%CI |
| --- | --- | --- | --- | --- | --- | --- |
| Age ≥50 | -0.83 | 0.335 | 6.126 | 0.013 | 0.436 | 0.226-0.841 |
| Postmenopausal | 0.985 | 0.303 | 10.555 | ＜0.001 | 2.678 | 1.478-4.825 |
| Gravidity ≥3 | -0.35 | 0.344 | 1.04 | 0.308 | 0.704 | 0.359-1.381 |
| Parity ≥2 | 0.958 | 0.379 | 6.396 | 0.011 | 2.606 | 1.24-5.474 |
| LEEP | 0.462 | 0.488 | 0.898 | 0.343 | 1.587 | 0.610-4.129 |
| Type III TZ | -0.559 | 0.292 | 2.033 | 0.154 | 0.571 | 0.265-1.233 |
| Multiple quadrants involved | 2.385 | 0.422 | 31.996 | ＜0.001 | 10.857 | 4.752-24.808 |

LEEP, loop electrosurgical excision procedure; TZ, transformation zone
